# Understanding the dynamic interactions driving the sustainability of ART scale-up implementation in Uganda

**DOI:** 10.1186/s41256-018-0079-6

**Published:** 2018-08-06

**Authors:** Henry Zakumumpa, Nkosiyazi Dube, Respicius Shumbusho Damian, Elizeus Rutebemberwa

**Affiliations:** 1 School of Public Health, Makerere University, Kampala, Uganda; 2 School of Health and Community Development, University of the Witwatersrand, Johannesburg, South Africa; 3 Faculty of Social Sciences, University of Dar es Salaam, Dar es Salaam, Tanzania

**Keywords:** Health systems, Sustainability, HIV treatment, Dynamic interactions, Systems thinking

## Abstract

**Background:**

Despite increasing recognition that health-systems constraints are the fundamental barrier to attaining anti-retroviral therapy (ART) scale-up targets in Sub-Saharan Africa, current discourses are dominated by a focus on *financial* sustainability. Utilizing the health system dynamics framework, this study aimed to explore the interactions in health system components and their influence on the sustainability of ART scale-up implementation in Uganda.

**Methods:**

This study entailed qualitative organizational case-studies within a two-phased mixed-methods sequential explanatory research design. In Phase One, a survey of 195 health facilities across Uganda which commenced ART services between 2004 and 2009 was conducted. In Phase Two, six health facilities were purposively selected for in-depth examination involving i) In-depth interviews *(n = 44)* ii) and semi-structured interviews (*n = 35*). Qualitative data was analyzed by coding and thematic analysis. Descriptive statistics were managed in STATA (v 13).

**Results:**

Five dynamic interactions in ART program sustainability drivers were identified; i) Failure to update basic ART program records contributed to chronic ART medicines stock-outs ii) Health workforce shortages and escalating patient volumes prompted adaptations in ART service delivery models iii) Broader governance issues manifested in poor road networks undermined ART medicines supply chains iv) Sustained financing for ART programs was influenced by external donors v) The values associated with the ownership-type of a health facility affected ART service delivery and coverage.

**Conclusion:**

The sustainability of ART programs at the facility-level in Uganda is a function of a complex interaction in elements of the health system and must be understood beyond sustaining international funding for ART scale-up*.*

## Background

The rapid expansion in anti-retroviral therapy (ART) coverage in Sub-Saharan Africa (SSA) has depended substantially on global health initiatives (GHIs) notably The Global Fund established in 2002 and The President’s Emergency Fund for AIDS relief (PEPFAR), established in 2003 [1].

In the broader Sub-Saharan Africa (SSA) region, and Uganda in particular, PEPFAR and The Global Fund have supported ART scale-up implementation in public and private health facilities since June 2004. This support was provided in form of provision of free antiretroviral drugs (ARVs), workforce training in ART management, enhancing laboratory capacity and strengthening ART program reporting [2, 3].

After years of sustained increases in global health aid for ART scale up implementation in SSA, recent indications point to a decline in international funding flows for ART scale-up [4, 5]. This decline in funding comes amidst the escalating demand for ART arising from adoption of the ‘universal test and treat’ policy which recommends that all diagnosed as HIV positive be enrolled on sustained ART regardless of disease stage [6, 7]. Of the 37 million living with HIV globally, only 18 million were enrolled on ART by mid-end of 2016 [8].

Against this background, the sustainability of ART scale-up implementation in SSA has come into critical focus [10, 11]. Despite increasing consensus that health-systems constraints are the fundamental barrier to attaining ART scale-up targets [12], current discourses on ART scale-up sustainability have been dominated by a narrow focus on *financial* sustainability [9, 10, 13]. Studies have been done modeling future HIV treatment needs based on unit cost analyses [14–17].

Despite calls by The World Health Organization (WHO) for applying a ‘systems thinking’ lens in understanding bottlenecks in scaling up public health interventions [18–21], fragmented approaches that focus on individual blocks of the health system, such as financing, persist. Indeed, there is a dearth of analysis situating the sustainability of ART scale-up implementation within a broader framework of the six ‘building blocks’ of a health system [22], and the dynamic interactions in these elements [23]. Moreover, Mounirer-Jack and colleagues [24] observe “the literature is relatively silent on the interactions between the six building blocks in the framework”.

**Fig. 1:**
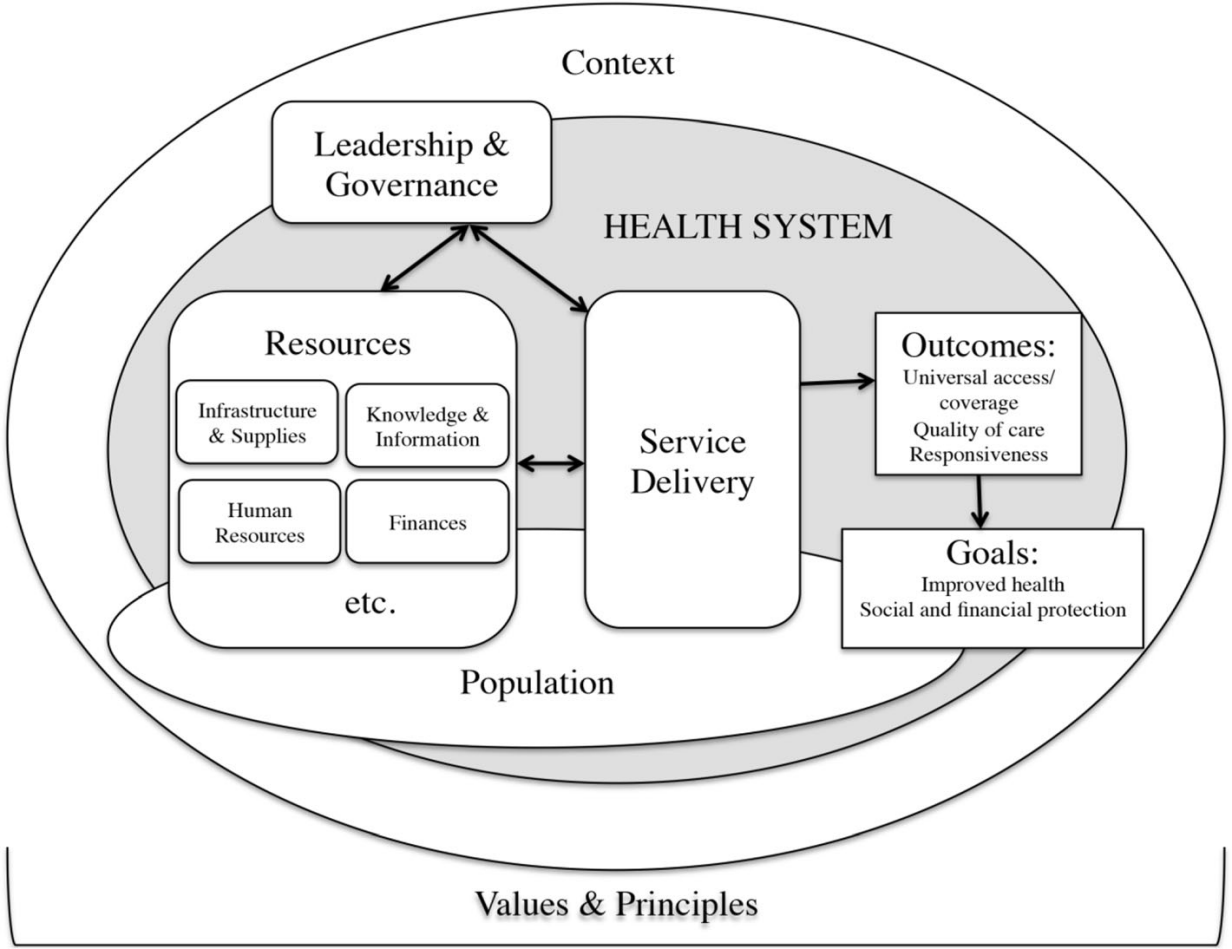
The health system dynamics framework by van Olmen et al. (2012) in [25]

This study is broadly informed by van Olmen et al.’s health system dynamics framework [25]. The van Olmen framework builds upon the earlier WHO’s building blocks framework to introduce 10 elements underpinning a health system [25, 26]. Beyond the six building blocks (Human resources, Finance, service delivery, governance and leadership, medicines, health information systems), Fig. 1 shows that the framework incorporates additional elements (such as ‘population’, ‘context’, ‘values & principles’) theorized as interconnected components in a health system. More pertinent to this study, the framework posits that these elements are not ‘mechanical’ blocks that are independent of each but that there is a complex dynamic interaction in the 10 elements underpinning a health system [25].

Utilizing the van Olmen framework, this paper aimed to explore how different health system components interact in influencing the sustainability of ART scale-up implementation at the level of health facilities in Uganda.

In contrast with studies probing ART scale up sustainability from the perspective of global health aid [27, 28], and studies focusing on domestic HIV spending [29, 30] as well as analyses at the micro-level [3, 31], this study focusses at the meso-level [32]. The meso level is at the level of organizations that make up a health system such as hospitals or other health care institutions which operate in the broader environment of local, national and global contexts.

## Methods

### Research design

The study documented in this paper is derived from a broader doctoral research project interrogating the sustainability of ART scale-up implementation in Uganda [5, 11, 33, 34, 35]. This entailed a two-phased mixed-methods sequential explanatory research design involving a national survey of health facilities (phase 1) and organizational case-studies (phase 2) [5, 11, 33, 34].

This paper draws primarily from qualitative data from phase 2 of the larger study. Core qualitative analyses of the organizational case-studies [36] were conducted supplemented by a quantitative component from the facility-survey in phase 1 of the study, part of which is reported elsewhere [5, 11, 33, 34]. Stirman and colleagues [37], in a seminal review of the literature on health program sustainability, found that qualitative methods were better suited to capturing the complex interactions in sustainability drivers which informed the priority assigned to qualitative analyses in this paper.

### Study sites and sample selection

#### Phase one

Health facilities which participated in the emergency phase of ART roll-out in Uganda [3] between 2004 and 2009 were selected in order to gain a long-term lens on ART scale-up implementation. To achieve this, health facilities were selected in a 2-stage process. In the first instance, we secured the published Uganda Ministry of Health, ART monitoring Unit Report of March 2010. According to this report, there were 394 accredited ART sites in Uganda at the end of March 2010. The list of accredited ART sites, contained in this report, served as the sampling frame for the study. Utilizing proportionate size to sampling method, a sample of 195 (out the 394) health facilities, which participated in the emergency phase ART roll-out, were randomly selected. The detailed sample selection criteria resulting in a nationally-representative sample of 195 health facilities across Uganda is described elsewhere [33] (Table 1).

**Table 1:** Representation by ownership-type of health facility

Type of ownership	National sample (%)^a^	Number of health facilities to sample
Public facilities	62	121
Private not- for- profit	18	35
Private for-profit	17	33
HIV research clinics	3	6
	100	195

^a^National sample based on the Ministry of Health March–June 2010 ART Monitoring Unit Report

#### Phase two

Phase Two adopted a multiple case-study approach [38]. Six health facilities were purposefully selected from 195 health facilities which participated in a survey [11, 35] to measure the extent of ART program institutionalization using level of institutionalization scales (LoIn). Health facilities with the highest scores (2) from the survey findings, those with the lowest scores (2), and those which did not sustain ART services (2) were purposively selected [11]. As Table 2 shows, the outcome of this selection resulted in a sample of six cases that were broadly reflective of the characteristics of ART-providing facilities in Uganda in terms of; a) Setting (rural/ urban), b) level of care [39] (tertiary, secondary, primary) and c) type of ownership (public, for-profit, not-for-profit).

**Table 2:** Health facility demographic information

Demographic features:	P-001	PNFP-001	P-002	PFP-001	PFP-002	PFP-003
Ownership-type	PUBLIC	NOT FOR PROFIT	PUBLIC	FOR-PROFIT	FOR-PROFIT	FOR-PROFIT
Level of care	Regional Referral Hospital	General Hospital	Health Centre IV	Health Centre III	Health Centre III	Health Centre II
Setting	Urban	Urban	Peri-urban	Urban	Rural	Rural
ART Clinic staffing strength	53	63	2	1	*	*
ART scale-up commencement	2004	2005	2006	2009	2009	2009
Number of patients (June 2010)	9540	2556	146	84	342	11
Number of patients (June 2015)	24,408	4337	458	19	0	0

*ART services discontinued in 2013

### Data collection

#### Phase one

Data were collected through a structured questionnaire which was self-administered by the head of the ART clinic at each of the 195 participating health facilities during the first half of 2014.

A 35-item questionnaire containing both closed-ended and open-ended items was used. The first section of the questionnaire sought to elicit data on the organizational characteristics of participating health facilities (e.g. ownership-type, rural/urban setting) and respondent demographics (e.g. Cadre of health worker, work experience). The rest of the questionnaire was structured around the three broad categories of factors proposed by Shediac-Rizkallah & Bone [9] as potentially influential on health program sustainability and the determinants therein: *Intervention characteristics* (e.g. Modifiability of service delivery models) 2. *Organizational context factors* (e.g. Human resources, Financing) 3. *Broader environment factors* (e.g. Context, population, and medicines supply chains). An initial questionnaire draft was refined and improved after being pre-tested among 12 ART clinic managers drawn from health facilities outside the study sample but with comparable characteristics.

During on-site visits by a team of investigators and research assistants, a hard-copy questionnaire was handed to the head of the ART clinic at each of the 195 participating health facilities. To address potential non-response bias, reminders were sent to respondents every 2 days by phone call. A completed questionnaire was collected by a research assistant, on-site, a week after the initial visit.

#### Phase two

An interview guide was constructed around the three broad themes proposed as influential on health program sustainability (*a) Intervention characteristics b) Organizational context c) Broader environment factors* [9]. The interview guide comprised of 16 questions. A sample of the questions posed include *1. What strategies did your health facility adopt to enable health workers cope with increased patient loads following ART scale-up implementation? 2. How did your health facility ensure that ART services were maintained beyond the initial implementation phase of 2004–2009?*

Face-to-face interviews (*n* = 79) were conducted with an interviewee nominated by the head of the ART Clinic based on ART program experience and selected informants (Table 3) from each of the six case-study facilities to elicit different organizational perspectives.

**Table 3:** Summary of participants for phase two of the study

Type of interview	Participant category	Number of interviewees
In-depth interview	Head of the ART Clinic	6
	Head nurse	6
	Human Resources Manager	4
	Clinicians	18
	TOTAL	44
Semi-structured	Head of the ART Clinic	6
	Finance Manager	6
	Strategy Director	3
	Clinicians	18
	TOTAL	35

We sought interviewees with the longest ART program experience at the case-study facilities who we recruited with the help of the head of the ART clinic. Interviews were conducted in English at the respondents’ office within the ART clinics and lasted approximately between 35 and 60 min. The interviews were conducted by the first author and four research assistants experienced in qualitative research. The research assistants were trained prior to data collection on the basics of the WHO building blocks of the health system framework.

During the interview, participants were asked to describe characteristics of their ART program such as the number of patients currently enrolled on ART and the range of HIV services offered. Interviewees were then asked to describe how the ART program at their site had evolved since the initial phase of ART roll-out. An open-ended approach was adopted at the start of the interview to understand what factors influenced the sustainability of ART service delivery from the perspective of interviewees. When items from the interview guide were not spontaneously raised, they were subsequently posed.

### Data analysis

#### Phase I

Close-ended responses to items in the questionnaire were edited, cleaned and initially entered into Epi Data (version 3.1). Data were later exported into STATA software (version 13) for analysis. Descriptive statistics were generated and used to compute percentages and frequency counts.

#### Phase II

We employed a thematic analysis approach suggested by Miles & Huberman [40]. The audio-recorded interviews were transcribed into text transcripts by the first author and later validated by two authors (DS, ND). As a first step, three authors (HZ, DS, ND) read the transcripts multiple times for data familiarization [40]. The three authors then separately coded the interviews utilizing a predominantly deductive approach to analysis [40]. The 10 domains proposed in the van Olmen framework [25] (Fig. 1), were adopted as the overarching thematic framework for data analysis [25]. In the second stage, the data coded separately by the three authors, based on an initial coding scheme derived from the adopted thematic framework of 10 domains, were discussed in joint sessions involving all three authors to seek convergence in the assignment of codes and themes in a consensus process that resolved disagreements [19]. After the coding and categorization of data based on the 10 elements of the health system, the third stage involved analysis aimed at exploring interactions between the 10 elements of the health system. In this third stage, a team-based process involving all authors [19] discussed the five emergent principal interactions between the 10 elements within our adopted framework [25]; *i) Infrastructure & Supplies AND Knowledge & Information ii) Human Resources AND Service delivery iii) Leadership & Governance AND Infrastructure & Supplies. iv) Financing AND Context v) Values & Principals AND Service delivery.*

A respondent validation workshop [19] was conducted with 12 ART clinic managers drawn from the six case-study health facilities. During the workshop, a focus group discussion was conducted to elicit feedback on the five emergent dynamic interactions. The feedback received informed the final analyses.

### Mixed-methods integration

We adopted a qualitatively–led mixed methods approach [41, 42]. Qualitative methods have been found to be better suited in capturing the ‘complexities’ of health systems [19, 31]. To this end, core coding and thematic analyses [43] were undertaken resulting in five thematic interactions in health system components. Descriptive statistics were used for expansion of the five emergent themes [36, 43]. Hence, the qualitative and quantitative data are integrated and presented under the five emergent themes presented in the results.

## Results

### Characteristics of the sample

A total of 195 health facilities participated in the study. With respect to ownership-type, 121 (62%) of the 195 health facilities were public, followed by not-for-profit facilities 35 (18%), and for-profit facilities 33 (16%) and HIV Research Clinics 6 (3%).

In terms of setting, 88 (45%) of the health facilities were located in peri -urban settings or urbanized parts of rural areas, this was followed by those located in urban settings 78 (40%) and those based in rural areas 29 (15%).

Table 4 shows that by level of care in the Uganda health system [39], the majority of facilities were health centre IVs or public facilities catering to an area equivalent to a county or sub,-district, these were followed by General Hospitals.

**Table 4:** Participating health facilities by level of care in the Ugandan health system [39]

Level of Care	Percentage	Number
Health Centre IV	36.9%	72
General Hospital	29.7%	58
Health Centre II	16.9%	33
Health Centre III	9.3%	18
Regional Referral Hospital	6.4%	12
National Referral Hospital	0.8%	2
	100%	195

### Characteristics of respondents

Overall, there were a total of 236 respondents. In terms of gender, 53% (125) of the respondents were male while 47% (111) were female. The mean work experience of respondents was 8 years (1–20).

Of the 236 respondents, 195 were heads of ART clinics, while 18 were clinicians and 23 were managers within the health facilities (e.g. Human Resources/ Finance managers).

### The dynamic interactions driving the sustainability of ART scale-up implementation

In keeping with van Olmen et al. [25] notion of interactions in components of the health system, the results are presented based on the dynamic interactions that emerged in the final analyses rather than as stand-alone blocks.

### Infrastructure & Supplies AND Knowledge & Information

#### ART program reporting and its influence on the supply of ART medicines

Of the 195 health facilities, 181 (63%) identified irregular and insufficient supply of antiretroviral drugs (ARVs) as a barrier to the sustainment of ART services in the last 6 years preceding data collection in 2014. In the in-depth analysis of the six case-study facilities, chronic ART medicines stock-outs were reported across the six health facilities. However, 4 (out of 6) health facilities reported more frequent ART medicines stock-outs (P-001, PFP-001, PFP-001, PFP-002, PFP-003).

*‘Drug stock outs of ARVs are chronic here. There is irregular supply of commodities and sometimes certain drug combinations are not in stock. Patients get fed up when we keep telling them ‘come back next week’ when we hope to have their combinations in stock’ [Interviewee 1, PFP-003].*

All four health facilities which reported more frequent ART commodity stock-outs, also reported a related constraint of failure to keep and update basic ART program records. These include a register of ART patients, WHO clinical staging system data for each patient and dispensing logs. Representatives from these four health facilities indicated they had low staffing levels and that the available workforce preoccupied with routine HIV services with little spare time for updating basic ART program records. This impeded the process of forecasting and ordering a sufficient stock of ARVs.

*‘We only have two staff, a clinical officer and orthopedic assistant as the core staff at our ART clinic. The number of patients can reach as high as 200 patients on an ART clinic day. They are too busy serving clients and as such we have been a little behind on manually filling out the ART program forms. They don’t even have time to do ARV drug forecasts and requisitions on time’ [Interviewee 1, P-002].*

Due to a centralized national HIV commodities supply chain management system in Uganda, health facilities periodically submit forecasts of their ART medicines and commodities needs to The National Medical Stores (NMS) or Joint Medical Stores (JMS). The paucity of ART program data, at the facility-level, was identified as an impediment to the accurate forecasting of commodity needs and was linked with frequent ARV stock-outs at 4 (of the 6) case-study facilities.

### Human resources and service delivery

#### Health workforce shortages as a driver of adaptations in ART service delivery models

Health workforce shortages were one of the most cited bottlenecks in sustaining ART scale-up implementation in participating health facilities. The shortage of physician-level cadre was identified as a debilitating constraint in ART service delivery across both the survey and the case-study. Adaptations to the traditional model of physician-intensive HIV care were reported in 181 out of 195 (93%) health facilities which indicated that non-physician cadre were prescribing anti-retroviral therapy. A range of non-physician cadre were reported to be involved in clinical management of ART. The survey results show that the category of health workers reporting the highest involvement in prescribing ART were clinical officers (33.9%), followed by nurses (29.7%), doctors (22.5%) and midwives (4.0%). Almost 10% of the workforce prescribing ART in the surveyed health facilities were not from a cadre of health workers recognized by the Ministry of Health of Uganda.

*“The patient numbers are big but we have trained our nurses. Our nurses help us to handle the stable clients. Nurses can help a great deal because patients need to see a doctor at least once or twice a year but they can see a nurse on any other visit to the clinic” [Interviewee 2, PNFP-001].*

In the six case-study facilities, however, it was revealed that community health workers as well as select ‘expert clients’ were engaged in clinical roles at PNFP-001, a mission general hospital and P-002, a sub-district public health Centre, respectively.

In the context of rapidly expanding patient volumes over the course of ART scale-up implementation (See Table 1), health facilities sought to decongest ART clinics by evolving a clinic appointments-system that catered to the needs of a diverse patient population. Contrary to the fixed patient review periods spelt out in Uganda’s national ART treatment guidelines, the ART clinic population at a mission hospital (PNFP-001) was divided into those deemed clinically stable and those not and this was used as a basis for determining the frequency of ART clinic appointments.

*‘The ART clinic population was divided into two. We have both facility-based and outreach services. Those clients who are clinically stable and live near the hospital those we visit in their homes. Those who are not stable attend the ART clinic more frequently’ [Interviewee 3, PNFP-001].*

The influential role of ‘demand side’ drivers as a dynamic within a health system (or ‘population’) under the van Olmen framework) is illustrated in this study. Table 1 shows that in a number of case-study health facilities, ART enrollments more than doubled between 2010 and 2014. In the survey, the majority of health facilities 168 (86%) indicated that ART patient volumes had increased in the last 6 years preceding data collection. The internal service delivery capacity of participating health facilities interacted with broader context factors particularly the escalating demand for ART.

*‘The clients are increasing and patients are now living longer. The numbers will only keep going up. However, the amount of funds received for ART programs is not increasing, the number of staff are not increasing but remaining the same or decreasing’ [Interview 2, P-001].*

*“People want the (ART) services but it’s tricky for me. Much as we were given donor support, there was a gap in human resources. Because if I had the staff, trained and competent, I would have the capacity to sustain ART services but because of the few staff I can’t do that. You get it?” [Interviewee 1, PFP-001].*

### Leadership & governance and Infrastructure & Supplies

#### The state of public infrastructure and its influence on ART commodity supply chains

Broader governance challenges of low investment in public infrastructure in Uganda manifested in poor road networks and irregular electricity supply were identified as barriers to sustaining ART service delivery especially at lower-tier, rural health facilities. Specifically, poor road networks were implicated as a barrier to securing ART commodity supply chains in case-study health facilities located in rural areas (PFP-002 and PFP-003).

Two affiliated for-profit health facilities based in rural Western Uganda in close proximity to each other described impassable roads in their locality as an impediment to sharing ARVs during drug stock-out events.

*‘We have a private clinic in our network of health facilities. Not very far from here. We could actually share ARVs with them because they are always well-stocked and they are part of our network of providers but the road linking us is terrible’ [Interviewee 1, PFP-002].*

At P-002, a sub-district health facility based in a peri-urban setting, unreliable electricity supply was identified as a barrier to ART service delivery. Intermittent electricity supply hindered the running of laboratory tests such as those for CD4 count and the storage of samples awaiting analysis.

The scenarios described above highlight the complex dynamic interaction between ART medicines supply chains with broader development challenges of public infrastructure that touch on the governance and leadership context in Uganda.

### Financing AND context

#### Financing for ART programs and external donor contexts

High levels of dependence on external funding for ART service delivery emerged strongly in our findings. Of the 195 health facilities, 183 (94%) indicated that a PEPFAR implementing organization as a major source of funding for ART service delivery.

*‘I have major questions on the sustainability of HIV services here because if the donors were to pull out and we were to depend on government entirely, we would not be able to provide services the way we are currently doing. For example, our project grant is running out and I have no idea how we will cope’ [Interviewee 3, P-001].*

Participants reported that they were dependent on external donors and that sustained external grants for ART service delivery were premised on achieving performance targets set by external donors such as increasing ART enrollments. PEPFAR, a leading source of funding for the national HIV response in Uganda, sets annual targets for all its beneficiaries in Country Operational Plans (COP). Out of the 195 heads of the ART Clinic surveyed, 119 (61%) acknowledged that external donor funding policies were influential on ART program continuation.

Compared to large tertiary hospitals which had advanced patient data systems (P-001, PNFP-001), lower-tier health facilities and small private clinics (P-001, PFP-002, PFP-003) were disadvantaged in competing for successor donor grants due to the paucity of ART program data.

*‘Donors come in and assess and see how we are doing based on the program data we maintain. How you are performing on SMC (safe male circumcision)? They come in and see, how are you doing? And if there is a gap, they come and address it’ [Interviewee 1, PNFP-001].*

Organizational leadership emerged as a key driver of ART program sustainability. Having a ‘program champion’ within a health facility was associated with ART program sustainability by 115 of the 195 (59%) heads of the ART clinic surveyed. Internal program champions were reported to foster ART program continuation through mobilizing supplemental funding for ART service delivery beyond the traditional funders such as PEPFAR and The Global Fund.

A major finding of this study is that sustained external funding was not a guarantee that health facilities would sustain ART programs in the long-term. Indeed, in all for-profit providers (3 out of 6) health facilities, ART services were discontinued at some point despite the availability of free ART commodities, medical equipment donations and on-site support supervision from an external funder. The absence of incentives to motivate the workforce to cope with rapidly expanded patient volumes brought on by ART scale-up at PFP-001, PFP-002, and PFP was implicated as a contributory factor to the discontinuation of ART services.

*‘We have a problem of high workload. As a human being you need to be financially motivated. The workload would not be a problem if some incentives are given to boost the morale of the overworked staff’ [Interviewee 3, PFP-002].*

### Values & Principals AND service delivery

#### Profit maximization and its influence on ART service coverage goals of providers

The values and principles associated with the ownership-type of a health facility affected ART service coverage. All 3 (out of 6) case-study health facilities which reported discontinuing ART services since the initial ART roll out, between 2004 and 2009, were for-profit clinics. Representatives from PFP-001, PFP-002 and PFP-003 indicated that they discontinued ART scale-up implementation, despite sustained donor support, because of a clash with their for-profit mission.

At PFP-002, the representatives reported that ART scale-up implementation dramatically increased their out-patient burden and depressed attention to non-HIV services such as the treatment of malaria and family planning services. The rapid increase in HIV patient volumes reportedly compromised their policy of ensuring patient privacy.

*‘Of course the majority who come to XXX (name of clinic) come here for privacy. When they can’t find privacy here, they leave. Sometimes they could enter my consultation room and I lock and we talk and I dispense’ [Interviewee 1, PFP-002].*

*‘People became used to getting free HIV services from here but the numbers overwhelmed us. It became too much for me so I had to stop. Even If I didn’t inform the Ministry of Health, I stopped.’ [Interviewee 3, PFP-003].*

Representatives from PFP-001 mentioned that profit maximization was a key organizational goal and that ART scale-up implementation frequently conflicted with the goal of making profits and catering to higher-tier patients as opposed to the broad base of patients attracted by the donor-funded ART services. As such, a deliberate policy of scaling-down on patient volumes and maintaining a cap at 20 patients was adopted at PFP-001.

*‘So my target was, let me enroll (patients on ART) but I shouldn’t go beyond a certain number. In fact I told my staff we shouldn’t go beyond 20 patients. So, I have to keep checking, how many do we have? Let us not go beyond this.’ [Interviewee 1, PFP-003].*

## Discussion

The discourse on the sustainability of ART scale-up implementation in Sub Saharan Africa has been dominated by a narrow focus on *financial* sustainability. We sought to reframe this discourse by embedding it within a broader health systems perspective as guided by the health system dynamics framework [25]. Our study demonstrates that there are multiple interactions in health system elements in driving ART program sustainability at the facility-level in Uganda. Five principal dynamic interactions emerged in our study; i) inability to update basic ART program records contributed to chronic ART medicines stock-outs ii) Health workforce shortages and escalating patient volumes prompted adaptations in ART service delivery models iii) Broader national governance challenges manifested in poor road networks undermined ART medicines supply chains iv) The financing of ART programs was influenced by external donor contexts and v) The values espoused by the ownership-type of a health facility (especially having a for-profit orientation) affected ART service delivery and coverage.

### Interconnections in health system sub-components

Our study provides empirical credence to theoretical notions of dynamic interactions in health system sub-components and fills a void in the literature identified by Mounier-Jack and colleagues [24] who note a dearth of literature illustrating interactions in the building blocks of the health system. This study contributes to the growing evidence demonstrating the utility of the health systems dynamic*s* framework as a tool of analysis within which to elicit these interconnections [6, 44, 45]. Our study findings challenge prevailing linear and reductionist approaches to health systems analysis [19, 31, 46] by highlighting the complex interactions in ART program sustainability drivers at the organizational or meso-level in Uganda.

### The dynamic interactions driving access to ART medicines

In this study, we found that access to ART medicines at the level of front-line health facilities was influenced by factors originating from three health system building blocks (information systems, human resources, governance & leadership). Specifically, that failure to maintain basic ART program records, owing to health workforce shortages, contributed to chronic ART medicines stock-outs. Rural-based facilities were unable to share ART medicines with their nearest, better-stocked counterparts, on account of the poor road networks linking them. Our results add to previous efforts which have highlighted the complex mix of factors impacting on access to medicines (ATM) [3, 44, 46]. A recent study in Uganda by Doherty and colleagues [6], found that infrastructure and supplies were one of the three most influential factors impacting on the implementation of Option B+ in Uganda within the 10 health systems elements proposed in the van Olmen framework. Although previous studies have documented the role of macro-level factors (e.g. drug thefts in central medicines stores) in accounting for point-of-care stock-outs [47, 48], this study’s contribution is in suggesting that part of the problem is ‘local’ or originating at the facility-level. The results presented here highlight facility-level impediments to securing a regular supply and sufficient stock of ART medicines. These linkages within the health system building blocks reported here underscore the importance of adopting ‘whole-of-system’ approaches by donors and program managers in Uganda and the broader SSA region in lieu of vertical approaches that focus on individual health system elements.

### ART service delivery models as a function of linkages between health system elements

Our findings demonstrate that the shortage of physicians in a context of rapidly increasing patient volumes compelled health facilities to devise modifications in ART service delivery models. This included adopting non-physician-intensive ART care models and scaling down on clinic-based care in favour of alternative platforms such as home-based care programs. In order to decongest over-flowing ART clinics, the patient population was divided into two based on those determined to be clinically stable and those not. Visits to clinics were reduced for those clinically stable and more frequent visits were permitted for those assessed to be unstable. Further research is needed to evaluate the potential for scale-up of the identified innovations in service delivery models. Our findings add to the mounting calls for implementation of differentiated care models for ART service delivery in resource-limited settings [7, 49, 50]. In a study evaluating the implementation of a health systems strengthening intervention in three districts in Zambia, Mutale and colleagues [51] found that the shortage of trained health workers negatively affected service delivery and undermined outcomes of implementing a complex health system intervention. A study by Ford and colleagues [52], in South Africa implicated a number of factors as influential on the sustainability of HIV treatment services in the Lusikisiki area of South Africa. Although they didn’t apply a systems thinking lens and utilized a case-study of a rural treatment centre in the Eastern Cape of South Africa, some of their findings agree with ours in as far as factors such as health workforce shortages and unreliable electricity impeded HIV service delivery.

### The broader contexts influencing ART program financing

Our study reveals that health facilities in Uganda are heavily dependent on external donors with 94% of the 195 health facilities reporting that a PEPFAR ‘implementing partner’ was a principal source of funding for ART service delivery. Due to the high levels of dependence on GHIs such as PEPFAR for financing ART programs, ART programming agendas including annual performance targets were donor-driven. Continued funding for ART programs was dependent on health facilities meeting priorities set by external donors. Of the 195 heads of ART clinics surveyed, 61% of them acknowledged that donor policies were influential on ART program continuation at their health facility. The reported levels of dependence on external donors for continued ART service delivery point to broader governance and leadership challenges particularly those relating to national priority-setting in Uganda. In this connection, calls for increasing country ownership of ART programs in Uganda and the broader Sub Saharan Africa region are warranted. Our study adds to calls for increased domestic spending on national HIV responses [3, 7], leveraging the growth in Uganda’s middle class by co-opting them in contributing to meeting the costs of HIV treatment [2, 53], introduction of innovative government levies to support HIV programs such as Uganda’s proposed AIDS Trust Fund to be financed through a levy on soft drinks [34]. Although numerous studies have focused on funding for ART scale-up from the perspective of GHIs [27, 28] and at the level of domestic government level [29, 30], our findings demonstrate that leadership at the level of ART-providing organizations plays a critical role in raising supplemental funding for ART programs thereby promoting long-term sustainability. The role of organizational leadership in fostering intervention sustainability, including through resource mobilization, is acknowledged in the literature [9, 54].

In their recent systematic review of research on health system challenges, Roncarolo et al. [55], found that although studies had been conducted on 8 of the 10 elements proposed in the van Olmen framework (Fig. 1), there were knowledge gaps with respect to two components namely ‘population’ and ‘context’. In this study, ‘population’ or ‘demand-side’ factors were manifested in the rapid increase in patient volumes over the course of ART scale-up implementation as well as being an in-put into the health system through the role of ‘expert clients’ in plugging health workforce shortages. Furthermore, we illuminate how ‘context’ factors influenced the availability of funding for ART programs at the meso-level. Hence, the findings presented here contribute to reducing the dearth of evidence with respect to ‘population’ and ‘context’ as identified by Roncarolo and colleagues [55].

We found that even when we categorized our findings under five principal interactions, multiple dynamic interactions in health system components were still discernable even within this grouping. For instance, with respect to the dynamic interaction between health information systems and access to ART medicines, health workforce issues were implicated as well. Specifically, shortage of health workers undermined the updating of basic ART program records which in turn hampered the forecasting and eventual supply of ART medicines leading to stock-outs. According to The WHO [56], the health system building blocks are “dynamic, complex entities such that examination of their interaction cannot be simplistic, single variable, linear analysis”. Furthermore, Paina and Peters [57] posit that “the interactions of system components are typically complex and non-linear, and are not easily controlled or predictable”. Our study lends empirical support to these theoretical notions by illuminating these complex interactions. This contrasts with previous studies which report findings of their studies with the building blocks of the health system as separate, independent compartments [24, 58].

### Implications for funders and ART program managers in Uganda and the broader SSA region

Our study findings have implications for ART program managers and policy planners in Uganda and other resource limited settings. In light of the finding that failure to update basic ART program records contributed to chronic ART medicines stock-outs, it is recommended that interventions aimed at strengthening ART program reporting systems especially at the primary care level in Uganda [39] be prioritized. Twining programs where higher-tier hospitals are paired with lower-level health facilities as well as prioritizing on-site supervision could strengthen ART program information systems and potentially reduce the number of stock-out events of ART medicines and is worthy of consideration by the ART programs monitoring unit of the Ministry of Health in Uganda.

The findings presented here have relevance to funders of ART scale-up implementation in Uganda and the broader Sub Saharan Africa region. We found that for-profit providers discontinued ART services despite availability of a free supply of ART medicines, regular trainings for ART clinic personnel and medical equipment support. Our findings highlight the importance of re-thinking fragmented approaches for supporting ART scale-up implementation that focus on individual health system components (such as ART medicines) in favour of adopting whole-of-system perspectives that engender synergistic approaches. Specifically, our findings illuminate health workforce incentives as the ‘missing link’ in ART scale-up implementation which previous studies has been recognized as an area that is often ignored by funders [1].

With respect to the debate on the sustainability of ART scale-implementation in SSA, our findings challenge the prevailing fragmented approaches, characterized by on over-emphasis on the financing of ART scale up at the expense of other component parts of the health system and adds to calls for a broader understanding of sustainability in the HIV response [10, 13].

### Limitations

Some limitations are important to acknowledge. This study utilized the mixed-methods sequential explanatory research design. Phase one involved a survey of a nationally-representative sample of health facilities in Uganda which participated in the first trial ART roll-out phase through random sampling. However, in Phase two, we purposively selected six health facilities from this national sample of health facilities for in-depth examination. Although this enabled us to gain an in-depth, contextualized, understanding of the interaction in health systems elements in influencing ART program sustainability, statistical generalizability of the findings from this study phase may be limited [19].

The health facilities selected for this study were drawn from those which participated in the pilot phase of national ART roll-out in Uganda. ART roll-out in Uganda commenced at a relatively higher level of care (tertiary /secondary) in the Ugandan health system between 2004 and 2009. Although our study sample was broadly representative of HIV service delivery characteristics during the latter period, the 195 health facilities may not be fully representative of current HIV service provision by level of care in Uganda following decentralization of HIV services to lower-level health facilities. In the organizational case-studies, we selected informants who had the longest ART program experience. Although this helped in selecting interviewees who had substantial experiences to draw upon and offered a rich source of information, the perspectives of those who have since resigned may not be fully represented by the longest-service ART clinic staff.

## Conclusion

The sustainability of ART programs at the facility-level in Uganda is a function of a complex interaction in elements of the health system and needs to be understood beyond sustaining external financing of ART scale-up*.* This study underscores the importance of holistic or ‘system-wide’ approaches in promoting the sustainability of ART scale-up implementation, as opposed to focusing on individual building blocks (such as financing). Further research is needed to evaluate the potential for scale-up of the identified innovations in service delivery models such as spacing ART clinic appointments and non-clinic- based care platforms in the context of declining international HIV funding. Health system strengthening interventions especially targeting lower-level and rural-based health facilities are recommended to promote ART program sustainability. The policy and programming implications of the study are also discussed.

## Abbreviations

AIDS: Acquired immune deficiency syndrome
ART: Anti-retroviral therapy
ARVs: Anti-retroVirals
ATM: Access to medicines
GHI: Global health initiatives
HIV: Human immunodeficiency virus
JMS: Joint medical stores
NMS: National medical stores
PEPFAR: the Presidents’ emergency fund for AIDS relief
SSA: Sub-Saharan Africa
WHO: World health organization
